# Epigenetic reprogramming of host genes in viral and microbial pathogenesis

**DOI:** 10.1016/j.tim.2010.07.003

**Published:** 2010-10

**Authors:** Konstantinos Paschos, Martin J. Allday

**Affiliations:** Section of Virology, Division of Infectious Diseases, Faculty of Medicine, Imperial College London, Norfolk Place, London W2 1PG, UK

## Abstract

One of the key questions in the study of mammalian gene regulation is how epigenetic methylation patterns on histones and DNA are initiated and established. These stable, heritable, covalent modifications are largely associated with the repression or silencing of gene transcription, and when deregulated can be involved in the development of human diseases such as cancer. This article reviews examples of viruses and bacteria known or thought to induce epigenetic changes in host cells, and how this might contribute to disease. The heritable nature of these processes in gene regulation suggests that they could play important roles in chronic diseases associated with microbial persistence; they might also explain so-called ‘hit-and-run’ phenomena in infectious disease pathogenesis.

## Background on epigenetics

**Figure 1:**
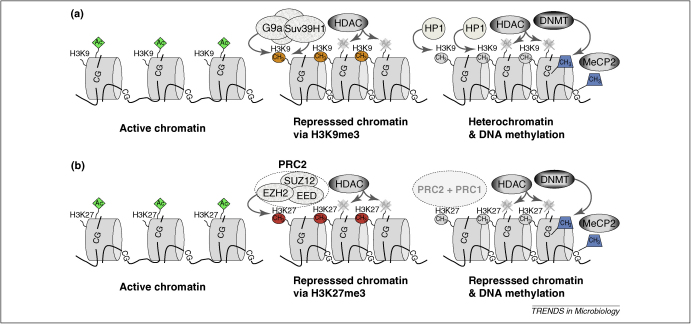
Histone modifications play at least two roles in the establishment of the epigenetic landscape of a cell. They can act as heritable repressive marks that compact chromatin and inhibit transcription of specific genes or loci, but they can also focus DNMTs to chromatin, thus establishing the more stable epigenetic mark CpG methylation. In general, regions permissive for transcription have ‘open’ active chromatin, marked by hyperacetylation of histones H3 and H4 at multiple sites and trimethylation of histone H3 at lysine 4 (H3K4me3, not shown). HDAC remove acetyl groups (Ac) from H3 and H4, and at least two complexes initiate further repressive histone modifications. **(a)** Complexes containing one or both of the histone methyltransferases G9a and SUV39H1 are responsible for trimethylation of H3 at lysine 9 (H3K9me3), establishing chromatin in a ‘closed’ repressive state. H3K9me3 then recruits the heterochromatin-protein HP1 to form heterochromatin; G9a, SUV39H1, HDACs and HP1 are all capable of binding and recruiting DNMTs, which can then facilitate CpG methylation ([89–92] and references therein). Methyl cytosines established by the DNMTs serve as docking sites for methyl-CpG binding domain (MBD) proteins such as MeCP2. **(b)** Polycomb-mediated repression facilitates a second repressive modification. PRC2 is composed of SUZ12, EED and the H3 lysine 27 methyltransferase EZH2, which establishes the H3K27me3 mark. H3K27me3 recruits PRC1, which includes BMI1 and RING1B and is responsible for the additional repressive modification of ubiquitinylation of histone H2A at lysine 119 (H2AK119Ub, not shown). Components of PRC1 (BMI1) and PRC2 (EZH2) and HDACs physically associate with DNMTs and also provide a platform for CpG methylation [4,6,82]. Again, MBD proteins such as MeCP2 bind and consolidate the repression or silencing.

An epigenetic trait was recently defined as ‘a stably heritable phenotype resulting from changes in a chromosome without alterations in the DNA sequence’ [1]. Such changes are mediated by chemical modifications to chromatin on both DNA and DNA-associated histones (see Glossary). Posttranslational covalent modifications to the flexible NH_2_ terminus (tail) of histones include methylation, acetylation, phosphorylation and ubiquitylation, and these are associated with the structural organization of chromatin and its transcriptional status. However, not all histone modifications are truly epigenetic, as very few satisfy the heritable part of the definition. To establish and mediate epigenetic memory, such modifications must be transmitted during DNA replication [1–3]. Methylation of cytosine in CpG dinucleotides (often referred to as DNA methylation) also contributes to the epigenetic status of a gene locus. When this occurs in a CpG island adjacent to a transcription initiation site, it is generally associated with repression or silencing of transcription. Histone modification, DNA methylation and the resulting reorganisation of chromatin are closely interlinked enzyme-driven processes that determine the transcriptional status of genes, gene clusters and noncoding RNAs such as micro ((mi)RNAs (Figure 1). Most of the epigenetic markers mentioned above are associated with transcriptional repression. Multiple additional covalent modifications to histones exist in parallel to these, resulting in a complex and context-influenced ‘histone code’ that dictates transcriptional state [2]. Because most of these modifications are not strictly epigenetic (i.e. heritable), and space constraints limit the scope of this review, they will not be considered further.

Epigenetic processes have been heavily implicated in the development of cancer, in which repression or silencing of tumour suppressor genes is remarkably common (Box 1) [4–6]. A reasonable hypothesis stemming from this is that pathogens associated with the development of cancer might initiate or influence the epigenetic processes of host cells, leading to epigenetic reprogramming. Evidence is accumulating that this is the case, and might be a widespread phenomenon [7]. In addition, pathogens might manipulate epigenetic processes to influence host responses associated with immunity and inflammation, and to contribute to other forms of chronic disease.

The aim of this paper is to briefly review examples of epigenetic control of host genes by viral and bacterial pathogens, and highlight how this could be relevant in disease pathogenesis.

## Viral manipulation of host epigenetic marks

As obligate intracellular parasites, viruses have developed numerous ways of hijacking cell processes to facilitate the completion of their life cycle and sometimes to evade the immune responses of their host. Viruses that cause persistent (often latent) infections are likely to benefit from heritable epigenetic changes in host transcription that produce an environment for their latent or persistent state without having to continuously express the initiating effectors [8]. Host genes involved in cell cycle progression, senescence, survival, inflammation and immunity are prime candidates as targets for such epigenetic control. We use two human γ-herpesviruses, Kaposi's sarcoma-associated virus (KSHV or HHV8) and Epstein–Barr virus (EBV), to illustrate how epigenetic manipulation of host cells probably contributes to latency and is likely to be involved in disease pathogenesis. Other less well-characterised examples will then be described.

### KSHV

KSHV has a distinct tropism for B lymphocytes, but it also infects endothelial cells. Similar to EBV, KSHV establishes life-long latency in humans, but unlike EBV, the specific cell type in which it remains latent is unknown. As the name indicates, the virus is associated with Kaposi's sarcoma, but also with primary effusion lymphoma (PEL) and multicentric Castleman's disease), two rare forms of B cell lymphoma [9]. KSHV encodes a latency associated nuclear antigen (LANA), and this protein is invariably expressed in latently infected cells and can interact with cellular DNA methyltransferases (DNMTs). Bacterially expressed glutathione s-transferase-LANA binds to DNMT1, DNMT3A and DNMT3B *in vitro*, but LANA appears to interact preferentially with DNMT3A *in vivo* [10]. LANA was identified by chromatin immunoprecipitation (ChIP) analysis at promoters of cell genes whose transcription was altered when LANA from a retrovirus was expressed in endothelial cells [10]. These included the promoter of the tumour suppressor gene encoding H-cadherin (*CDH13*), a gene that is methylated in several types of cancer [11]. The *CDH13* regulatory locus was also substantially methylated in KSHV-positive BCBL-1 PEL-derived cells, so it is possible LANA recruits DNMT3A and initiates *de novo* DNA methylation in B cell lymphomagenesis [10]. It remains unclear how LANA is targeted to *CDH13* (or other repressed promoters), but its ability to interact with other chromatin modifying proteins might provide clues. The capacity of LANA to associate with the corepressors mSin3, SAP30 and CIR [12], and in particular the histone H3 lysine 9 (H3K9) methyltransferase SUV39H1 [13], is consistent with LANA playing a role in transcriptional repression via histone modification, which could precede DNA methylation (Figure 1). Moreover, the ability of LANA to bind heterochromatin protein (HP)1 and the methyl CpG binding protein MeCP2 as well as DNMTs [10,14,15], suggests that it might coordinate repressive histone modifications with DNA methylation.

More recently it was reported that LANA also binds to the promoter of the gene encoding the transforming growth factor (TGF)-β type II receptor (TGF-βRII) leading to DNA methylation and downregulation of transcription [16]. This epigenetic repression resulted in abrogation of TGF-β signalling, which could be rescued by ectopic expression of TGF-βRII. Because TGF-β signalling is generally anti-proliferative and/or pro-apoptotic in B cells [17], LANA probably influences latency and lymphoma development by epigenetic disruption of this pathway and concomitant enhancement of cell survival. Treatment with the DNMT inhibitor 5-aza-2-deoxycytidine increased TGF-βRII expression and made KSHV-positive PEL cells susceptible to TGF-β-mediated growth arrest and apoptosis, suggesting a novel approach to therapy [16].

Furthermore, an investigation into the roles of KSHV encoded miRNAs revealed that ectopic expression of miR-K12–4-5p1 could indirectly increase the levels of DNMT1, DNMT3A and DNMT3B mRNAs [18]. Because the latent state of the viral episome requires DNA methylation, it was suggested this increase in DNMTs is necessary to maintain viral latency, but of course the increased DNA methyltransferase activity could also facilitate CpG methylation of host cell genes such as *CDH13* and *TGF-βRII*. Therefore, it is possible that the interactions of KSHV factors with host chromatin modifiers and the epigenetic reprogramming of infected cells is also associated with a requirement of KSHV to epigenetically regulate its own genome.

### EBV

EBV is associated with several human tumours of B cell, T cell and epithelial origin, including EBV-associated Burkitt's lymphoma (BL), post-transplant lymphoproliferative disease, diffuse large B cell lymphoma, some forms of Hodgkin's lymphoma, and the epithelium-derived nasopharyngeal carcinoma (NPC) and gastric carcinoma (GC) [19,20].

*In vitro*, EBV can very efficiently induce the activation and continuous proliferation of resting human B cells. The resulting lymphoblastoid cell lines (LCLs) carry the viral genome as extrachromosomal episomes, and express only nine latency-associated EBV proteins. These include six EB nuclear antigens (EBNAs 1, 2, 3A, 3B, 3C and LP) and three latent membrane proteins (LMP1, 2A and 2B), and there are also several untranslated RNA species [20,21]. As we shall see, several of these proteins are probably involved in epigenetic modification of the host genome.

Data on the persistence of EBV in humans are consistent with the viral genome residing long-term in a resting memory B cell population. It is now considered probable that to establish persistence, EBV initially infects resting (naive) B cells and drives these to proliferate as activated B blasts. This expanding infected B blast population then migrates into germinal centres, where they differentiate to become centroblasts, centrocytes and finally resting memory B cells. Although the precise series of events that the EBV-positive B cells undergo to reach the memory compartment is unknown, it appears to involve regulated shutdown of latent protein expression from an initial state called latency III (as found in LCLs), via latency II (where only LMP1, LMP2 and EBNA1 are expressed) until latency 0, a state in quiescent memory B cells in which no EBV proteins can be detected. Occasionally memory cells divide and EBNA1 is transiently expressed to ensure maintenance of the viral episome (a state known as latency I) [19,22].

Because evidence indicated that expression of EBNA3 in BL cells dramatically enhanced their survival potential [23,24], recombinant EBNA3 knockout (KO) viruses were used to explore regulators of apoptosis that might be deregulated by EBV and contribute to this phenotype. This identified the gene encoding BIM *(BCL2L11)* as a target that is repressed by latent EBV through a mechanism requiring the functional cooperation of the EBNAs 3A and 3C [25]. BIM is a potent inducer of apoptosis and a crucial regulator of lymphocyte survival. Reduced expression of BIM enhances lymphomagenesis in mice and humans, and plays an important role in the pathogenesis of BL [26].

Subsequently, it was shown that EBV-mediated repression of *BIM* transcription initially involves the epigenetic modification H3K27me3 (histone H3 trimethylated at lysine 27), probably through polycomb repressive complex (PRC)2, a known mediator of this modification in humans (Figure 1, Glossary). The binding of PRC2 to the *BIM* promoter and trimethylation of H3K27 might then be followed by DNA methylation of sites within the CpG island that flanks the *BIM* transcriptional initiation site [27]. H3K27me3 has recently been confirmed as a *bone fide* heritable, repressive histone modification that often precedes DNA methylation in cancer (Figure 1, Box 1). Therefore, a working hypothesis is that EBV can epigenetically reprogram B cells and their progeny in this way, so that they are more prone to become cancerous, which suggests a model of how the EBNA3s might contribute to the pathogenesis of BL [19,25,27,28]. Briefly, because preventing the induction of BIM by the translocated and deregulated proto-oncogene *MYC* can be a crucial event in the development of BL, repression of *BIM* transcription by EBNA3A and 3C is likely to make an important contribution to the progression of EBV-positive BL. Furthermore, because newly infected B cells express EBNA2, which constitutively activates the expression of MYC [29], EBNA3A and EBNA3C might have evolved this repressor activity to prevent EBNA2/MYC-induced, BIM-mediated apoptosis during the establishment of normal EBV latency and persistence.

Consistent with the *BIM* study linking EBV with the polycomb system, it was recently shown that EBNA3A and EBNA3C also functionally interact in polycomb-mediated repression of the tumour suppressor gene encoding the cyclin-dependent kinase inhibitor p16^INK4A^ [30]. This repression of *p16*^*INK4A*^ not only overcomes cell cycle arrest and senescence in EBV infected B cells, but might also pave the way for DNA methylation of *p16*^*INK4A*^ control elements during lymphomagenesis.

*BIM* and *p16*^*INK4A*^ are not the only reported cases of epigenetic control by EBV. In epithelial cell clones expressing LMP1, increased DNA methylation associated with repressed transcription was found at the promoter of the E-cadherin gene (*CDH1*) [31]. Similar to H-cadherin, E-cadherin is an adhesion molecule that affects tumour invasiveness and is frequently epigenetically repressed in human carcinoma [5,32]. The increased DNA methylation was apparently associated with LMP1-mediated induction of DNMT1, DNMT3A and DNMT3B, probably via the c-Jun N-terminal kinase 1 pathway. However, it was not reported whether this general increase in DNA methyltransferase activity led to the CpG methylation of other tumour suppressor genes, nor whether LMP1-mediated repression of E-cadherin expression is a common feature of NPC. Also in the epithelial cell context, GC tumours carrying latent EBV show increased promoter methylation of genes encoding the p16^INK4A^, p14^ARF^, p73, E-cadherin and PTEN tumour suppressors, relative to EBV-negative GC. It has been suggested that these epigenetic changes could be caused by elevated levels of DNMT1, which in these cells might be induced by the expression of EBV LMP2 ([33] and references therein).

Generally, it seems that EBV has established epigenetic strategies to alter host gene expression to facilitate its life cycle. In B cells, EBNA3 proteins enhance cell survival and proliferation. In epithelial cells, the LMPs appear to be more important. In each case, the infected cells appear more likely to undergo malignant transformation.

### Hepatitis B virus

Hepatitis B virus (HBV) is associated with hepatocellular carcinoma (HCC), and several studies have indicated that the presence of HBV in HCC correlates with aberrant DNA methylation of the host genome [34–37]. HBV infected cells and HCC tumours show elevated expression of DNMT1, DNMT3A and DNMT3B relative to uninfected cells and matched normal tissues, respectively [38]. Because overexpression of HBV X antigen (HBXAg), another nuclear oncoprotein, can induce DNMT1 and DNMT3A [39,40], it has been suggested that this viral protein is responsible for what has been described as a ‘methylator’ phenotype in HBV-positive HCC. Consistent with this hypothesis, HBXAg can repress transcription from and initiate the CpG methylation of E-cadherin and p16^INK4A^ gene regulatory elements [35,41,42]. In both cases, this repression is thought to be a consequence of HBXAg-mediated induction of DNMT1.

### Human papillomavirus

There are over 100 types of human papillomavirus (HPV) and several, such as HPV16 and 18, are associated with the development of malignancies that include cervical carcinoma. It has recently been reported that a keratinocyte cell line carrying HPV16 episomes has reduced levels of E-cadherin [43]. The oncoprotein E7 was essential for this downregulation, which was reversible after treatment with the DNMT inhibitor 5-aza-deoxycytidine [43]. Although the precise mechanism was not described, E7 was shown to modulate DNMT1 levels [43] and has previously been shown to bind directly to DNMT1 and precipitate DNA methyltransferase activity [44]. In addition, because it disrupts the Rb-E2F pathway, E7 can induce expression of the H3K27 methyltransferase EZH2 [45]. Increases in EZH2 have been reported in several types of cancer, and might be associated with the DNA methylation of polycomb target genes (Box 1). It is therefore intriguing that p16^INK4A^, which is normally repressed in cycling cells by EZH2 via H3K27me3 and frequently undergoes DNA hypermethylation in cancer, is often overexpressed in HPV-positive carcinoma [4,46,47].

The presence of HPV in cervical carcinoma-derived cells also correlates with DNA hypermethylation of CpG islands at the 3.3 kb repeats in the subtelomeric regions of multiple chromosomes [48]. In particular DUX4, which is encoded by one of these regions (4q35), is downregulated in a significant percentage of HPV-positive cells [48]. DUX4 is a proapoptotic protein when overexpressed in cells, so it could act as a tumour suppressor [49].

HPV in low-grade squamous intraepithelial lesions is associated with increased DNA methylation of the promoters of genes encoding two putative tumour suppressors (*BLU* and *RASSF1*) that are adjacent (head to tail) on the genome [50]. It is not clear how these promoters are hypermethylated or what consequences result, but (RAS association domain family protein (RASSF)1 is a tumour suppressor that is silenced in many tumours by DNA hypermethylation [51].

### Simian vacuolating virus 40

Simian vacuolating virus (SV)40 is a polyomavirus that infects monkeys and humans, and is another oncogenic virus suspected of inducing epigenetic changes in host cells. SV40, similar to HPV, is associated with *RASSF1* promoter DNA methylation in malignant mesothelioma, where the presence of SV40 large T-antigen (Tag) sequences significantly correlates with high levels of DNA methylation [52]. The same correlation was not observed between infection and *RASSF1* methylation in small cell lung cancer, where this promoter is also sometimes hypermethylated [53].

Nevertheless, more specific sites of aberrant DNA methylation might await discovery in SV40 infected cells, possibly because expression of large T antigen also upregulates DNMT1, with concomitant increases in DNA methyltransferase activity and genomic methylation [54]. When 10 different tumour suppressor genes were examined in 90 cases of human lymphoid and hematopoietic malignancies, the presence of SV40 was found to correlate with promoter DNA methylation of seven of those genes, including p16^INK4A^, p15^INK4B^ and p73 [55]. However, apart from increasing the level of DNMTs, the mechanism of SV40-associated CpG methylation of cellular genes is not presently known.

### Adenoviruses

Although adenoviruses generally exhibit a lytic rather than latent life cycle, the viral oncoprotein small e1a has been reported to globally restrict histone 3 lysine 18 acetylation (H3K18Ac) and repress transcription of multiple genes [56,57]. However, it remains to be determined whether this imparts any sort of epigenetic memory or leads to more stable repression such as DNA methylation in infected cells or their progeny.

### HIV and human T cell leukaemia virus-1

More than a decade ago it was reported that infection of CD4+ T cells by either HIV-1 or human T cell leukaemia virus (HTLV)-1 results in an increase in cellular DNA methyltransferase activity [58]. This produced a general increase in cellular DNA methylation and a specific increase in methylation at the interferon--γ promoter, associated with transcriptional repression. Although it is still unclear what (if any) contribution this makes to HIV-associated disease, in HTLV-1-carrying adult T cell leukaemia/lymphoma (ATLL) many of the ‘usual suspect’ tumour suppressor genes, including those encoding p16^INK4A^ and p73, become hypermethylated [59]. This ‘methylator’ phenotype is associated with disease progression and crisis, but the mechanism has not been identified, so it remains to be determined whether there is a link with the induction of DNMT activity by HTLV-1 [58,59].

HIV infection of T lymphocytes in culture also causes promoter methylation of the gene *GNE*, which encodes a key enzyme in sialic acid biosynthesis, and represses its transcription [60]. This might lead to disruption of cell surface sialylation, which is important in cell–cell recognition and might have important effects on lymphocyte trafficking.

### Paramecium bursaria chlorella virus

Although *Paramecium bursaria* chlorella virus (PBCV)-1 infects *Chlorella* green algae and would probably not be considered a typical pathogen, it is worthy of consideration because it carries a gene encoding a viral SET (vSET) domain protein ([61] and references therein). This highly conserved domain is found in many eukaryotic proteins that associate with chromatin, including members of the polycomb protein group, and has histone lysine methyltransferase activity [62]. It was revealed that infection with PBCV-1 significantly increased methylation of histone H3K27 in its host and rapidly repressed genome-wide transcription [61]. Perhaps more remarkable, when vSET was expressed in human HeLa cells, it catalysed H3K27 trimethylation after the depletion of EZH2 by siRNA [61]. vSET-mediated methylation of H3K27 then promoted the recruitment of PRC1 on to chromatin, further demonstrating the conserved nature of the system. Surprisingly, to date, this is the only viral histone lysine methyltransferase to be identified.

## Bacterial modification of the host epigenome

Some chronic bacterial infections are also associated with malignancy, the most well-known and widely studied being *Helicobacter pylori* infection of human gastric mucosae (see below). Moreover, many microbes have evolved ways of eluding the immune response and, again, epigenetic changes in host cells have been implicated in these processes.

### Chlamydophila pneumoniae

An obligate intracellular pathogen that causes acute respiratory diseases, *C. pneumoniae,* similar to PBCV-1, encodes a SET domain protein. The chlamydial SET domain protein directly interacts with chlamydial histone H1-like proteins Hc1 and Hc2, and has a histone methyltransferase activity capable of methylating murine histone H3 *in vitro* [63]. Its role in pathogenesis has not been described. However, genomic DNA sequence analysis has revealed that a significant number of bacteria have SET domain genes [63,64], which could presumably have functions linked to chromatin modelling. However, because not all will enter the host cell nucleus, it is probable that they methylate non-chromatin proteins [65]. One can only speculate that these might also interfere with host responses and modulate pathogenicity.

### Campylobacter rectus

Experiments in which mice were orally infected with *C. rectus* revealed bacteria translocated to the placenta from the original site of infection in 46.7% of the cases. Concurrently, downregulation of the *Igf2* gene in the placenta was found to be associated with higher levels of DNA methylation at its promoter [66]. *Igf2* is an imprinted gene that is implicated in placental development and fetal growth [67]. It is known that oral infection with C. *rectus* can lead to increased risk of preterm births [68], and it will therefore be interesting to discover whether this involves bacteria-mediated epigenetic modifications to the host genome.

### Anaplasma phagocytophilum

This Rickettsiales intracellular pathogen replicates in human neutrophils and their bone marrow progenitors. In these cells it has to survive despite the presence of the multiple antimicrobial mechanisms employed by these primary defence cells, mechanisms mediated by a number of so-called ‘defence genes’. In a recent report that assessed the expression of 23 defence genes after infection of a monocytic cell line with *A. phagocytophilum*, 19 were downregulated [69]. Histone deacetylase (HDAC)1 expression was increased in the infected cells, as was its association with the promoters of most of the affected genes. There was evidence of histone H3 deacetylation at these sites, and although there was a concomitant increase in H3 methylation, it is not yet clear whether stable epigenetic markers become established [69]. It is now emerging that modulation of histone acetylation on host defence genes might be a relatively common feature of bacterial infections, as several bacteria, including *Listeria monocytogenes, Clostridium perfringens, Streptococcus pneumoniae* and *Mycobacteria* spp., can alter host histone acetylation and/or HDAC activity [69,70]. Is it possible that epigenetic reprogramming of defence genes is a widespread phenomenon?

### H. pylori

Infection with this Gram-negative bacterium can result in chronic gastritis and peptic ulcers, and chronic *H. pylori* infection is closely associated with intestinal metaplasia and the development of gastric cancer [71,72]. In a recent study, 25 genes and several repetitive DNA elements were tested for DNA methylation in 212 tissue samples from gastric lesions, with or without *H. pylori* infection [73]. There was a good correlation between infection and DNA methylation at CpG islands in chronic gastritis, and there was also a correlation between the amount of DNA methylation and cancer progression. However, the correlation between the presence of *H. pylori* and cancer progression was not entirely consistent [73]. This could be because DNA methylation caused by infection in chronic gastritis does not always lead to carcinogenesis, or because the heritable nature of the epigenetic changes renders the continual presence of the infection unnecessary.

Co-cultivation of human gastric cell lines with *H. pylori* induced DNA methylation of *CDH1*, the E-cadherin gene [74]. The effect is probably indirect and thought to be through the action of the inflammatory cytokine interleukin-1β, which is known to induce CpG methylation and is upregulated by *H. pylori* [74,75]. Another recent study using a small animal model also indicated that it is the infection-associated inflammatory response, rather than *H. pylori* itself, that is responsible for the induction of aberrant DNA methylation in cells of the gastric mucosa [76]. These data are consistent with the demonstration that polycomb target genes preferentially undergo aberrant DNA methylation during chronic inflammation of the intestine and in intestinal cancer (Box 1) [77].

It might be significant that all the bacteria that are most clearly associated with stable epigenetic modifications of host genes (e.g. *C. pneumoniae, C. rectus,* and *H. pylori*) are also linked to chronic disease.

## Concluding remarks and future directions

It is easy to understand from the examples of persistent viral infections (e.g. KSHV and EBV) how the ability to epigenetically influence host gene expression could have evolved and how it might play a major role in the pathogenesis of chronic disease. Similarly, although the mechanisms are less well understood, the idea of epigenetic changes in host cells associated with chronic bacterial infections (e.g. *H. pylori*) contributing to disease is conceptually satisfying. What is less obvious is the significance of such heritable changes in host cell behaviour during acute pathogen-mediated disease. Perhaps a unifying theme is the modulation of the immune response, inflammation and intracellular host defences. This requires further exploration.

In summary, from data available currently, several themes are emerging.

1. Latent viral infection can (de)regulate patterns of repressive histone modifications that could then precipitate aberrant DNA methylation and the reprogramming of infected cells and their progeny. This can involve the repression of tumour suppressor genes, for which there will be strong selection in the development in cancer. Examples include KSHV and EBV.
2. Infection by diverse viruses appears to induce expression of DNMTs and/or polycomb group (PcG) proteins such as EZH2. Examples include KSHV, EBV, HBV, HIV-1, HTLV-1, HPV, SV40 and adenoviruses. The important caveat with these data is that the expression of DNMTs and EZH2 correlates with cell proliferation rate; therefore a high level of expression might be a consequence of the proportion of cells proliferating in tumours and/or chronically inflamed tissue.
3. At least one virus, PBCV-1, and perhaps many bacteria, including *C. pneumoniae,* [63,64] encode SET domain proteins that could methylate histones and so alter the organisation of host chromatin. However they might target many non-histone proteins for methylation.
4. Stimulation of host HDAC activity might be a common feature of microbial infection. This could contribute to the epigenetic repression of ‘defence genes’ in host cells. The possibility that this occurs in non-infected bystander cells responding to the uptake of soluble factors is also worth exploring.
5. Inflammation, whether or not it is induced by infection, can be a potent initiator of aberrant methylation of PcG target genes. Therefore chronic inflammation might predispose cells to malignant transformation.
6. The stable heritable nature of epigenetic change implies that the initial inducers of transcriptional repression and chromatin modification need only be expressed transiently. Although in most cases, effectors are continuously present, this provides a rational explanation for potential ‘hit and run’ mechanisms in infectious disease pathogenesis.

For many pathogens, it is very difficult to make even educated guesses about the extent or the mechanics of epigenetic change they might induce. Currently available data largely provide snapshots of what is happening to ‘usual suspect’ host genes after infection. More comprehensive global studies are now necessary and possible.

We are now in the era of epigenetics [78,79], and technologies for global analyses are being developed at a remarkable pace. Various methods are available for determining genome-wide DNA methylation signatures [80] and methods such as ChIP–chip (ChIP, coupled with expression microarray technology) and ChIP-seq (ChIP, coupled with deep-sequencing analysis) for mapping global chromatin modifications are now well established [81]. Over the coming months and years, these approaches will be invaluable for exploring the contribution that infections make to the epigenetic landscape and demonstrating the dynamic nature of the human ‘epigenome’.

## Note added in proof

While this article was in the final stages of preparation, Soria *et al.* (Nature 466, 1076–1081, 2010), showed that the E4-ORF3 protein of oncogenic adenoviruses induces widespread epigenetic silencing of tumour suppressor p53-target genes.

## Box 1 PcG proteins and DNA methylation: cancer, aging and inflammation

PcG proteins are transcriptional repressors that play crucial roles in development and differentiation (Figure 1). Stem cells and cancer cells often share gene expression patterns, and PcG proteins are thought to be essential for the maintenance of stem cells and in oncogenesis [4,6,82]. Multiple reports suggest that PcG complexes contribute to the aberrant methylation profiles that are crucial in the genesis and progression of diverse cancers [83–85]. The mechanism for this is suggested by the capacity of PcG proteins to physically interact with DNMTs and recruit them to chromatin (Figure 1). It has been estimated that PcG target genes are up to 12 times more likely to become aberrantly methylated than non-targets [85]. However, it should be noted that polycomb-mediated gene silencing in cancer can also be independent of DNA methylation, and that DNA methylation is not an inevitable consequence of polycomb-associated histone modifications [86].

In *Drosophila*, repression by PcG spreads from polycomb response elements (PREs) located in a genomic regulatory locus, but the direct equivalents of PREs have not been identified in mammalian cells. The mechanism by which PcGs are targeted to specific chromatin loci is unknown and is a key problem in mammalian biology. The most likely candidates for PcG recruiting factors are sequence-specific transcription factors and/or long noncoding RNAs, either of which could be deregulated in cancer or other chronic diseases [4,6,82,87].

Recent reports indicate that both normal ageing and chronic inflammation contribute to the aberrant methylation of PcG target genes that are generally suppressed in stem cells [77,88]. We propose that in some circumstances pathogens, acting directly or indirectly, might have a similar effect.

## Glossary

Chromatin immunoprecipitation (ChIP): central to the study of epigenetic modifications, this is a technique that allows the specific precipitation of DNA sequences associated with chromatin-bound proteins, notably histones with specific epigenetic modifications. There are now hundreds of widely available verified antibodies that can be used for ChIP, which are also available as standardized ChIP kits.
CpG island: a region of DNA that contains the sequence CG more frequently than predicted in a random distribution of bases. When they are at the promoter of active genes, they are largely protected from DNA methylation.
CpG DNA methylation: the addition of a methyl group at position 5 of the aromatic ring in cytosine, which can occur when a cytosine is followed by a guanine in the DNA sequence. Generally associated with transcriptional repression, especially when found at gene promoters.
Deep sequencing: the concurrent sequencing of millions of molecules from a DNA library. The method is based on producing separate clusters of amplified, individual molecules for the original library, in single stranded (ss)DNA form. Nucleotides are incorporated onto ssDNA, according to DNA complementarity, which produces a light signal that can be read by an instrument. Successive rounds of incorporation allow reading the sequences of millions of clusters at the same time, according to the light signals emitted at each cluster position.
DNA methyltransferase (DNMT): enzymes mediating CpG DNA methylation. In humans, DNMT3a and DNMT3b are *de novo* DNMTs. DNMT1 is considered responsible for the maintenance of DNA methylation patterns because it is preferentially targeted to DNA that is methylated on one strand (hemimethylated) after replication to restore methylation on the newly synthesized strand.
Histone: Histones are proteins comprising the basic chromatin structural unit, the nucleosome. Histones H2A, H2B, H3 and H4 form an octamer (two copies of each) core, around which DNA winds 1.7 times to form the coiled nucleosome structure. Histones of the histone core have two functional domains, the characteristic histone fold that is necessary for histone–histone/DNA interactions and an NH_2_-terminal tail that protrudes from the nucleosome core.
Histone deacetylase (HDAC): enzymes mediating deacetylation at various lysine residues of histone tails. There are three classes of HDACs: class I and II HDACs are zinc-dependent and class III HDACs are NAD-dependent. Most of these enzymes do not show high preference for specific lysine residues.
Polycomb group (PcG) proteins: these were first identified in *Drosophila* and are best known as repressors of the homeotic (Hox) transcription factor genes during embryonic development, from flies to humans. They form multiprotein complexes called polycomb repressive complexes (PRCs) that bind and epigenetically regulate hundreds of genes, predominantly associated with cell-fate decisions and development.
Polycomb repressive complex (PRC)1: a complex that mediates the repressive ubiquitination at lysine 119 of histone H2A (H2AK119Ub). Core proteins include chromobox proteins, whose chromodomains are thought to recruit the complex to the H3K27me3 mark, and RING finger proteins, such as RING1B and BMI1, which are responsible for the E3 ubiquitin ligase activity of the complex at H2AK119.
Polycomb repressive complex (PRC)2: a complex that mediates trimethylation at lysine 27 of histone H3 (H3K27me3). In humans the core complex comprises three polycomb proteins: suppressor of zeste (SUZ)12, embryonic ectoderm development (EED) and enhancer of zeste (EZH)2. EZH2 contains the catalytic SET domain core, whereas both SUZ12 and EED are required to maintain complex integrity and the enzymatic activity of EZH2.
SET domain: protein domain responsible for lysine methyltransferase activity in many proteins. It is found in proteins of organisms as diverse as archaea, bacteria and humans, possibly as a result of horizontal transfer. EZH2, which catalyses H3K27 methylation, and G9a and Suv39H1, which catalyse H3K9 methylation, are examples of proteins with SET domains.
